# The possible regulatory role of miR-4463 and its target gene *CYP19A1* on the ovarian response in the women with diminished ovarian reserve: A case-control study

**DOI:** 10.18502/ijrm.v22i8.17237

**Published:** 2024-10-14

**Authors:** Azam Yazdanian, Marzieh Lotfi, Fateme Montazeri, Saeideh Dashti, Mohammad Hasan Sheikhha

**Affiliations:** ^1^Abortion Research Center, Yazd Reproductive Sciences Institute, Shahid Sadoughi University of Medical Sciences, Yazd, Iran.; ^2^Department of Genetics, Faculty of Medicine, Shahid Sadoughi University of Medical Sciences, Yazd, Iran.; ^3^Research and Clinical Center for Infertility, Yazd Reproductive Sciences Institute, Shahid Sadoughi University of Medical Sciences, Yazd, Iran.

**Keywords:** Ovarian response, CYP19A, miR-4463, Diminished ovarian reserve (DOR), Cumulus cells (CCs).

## Abstract

**Background:**

Diminished ovarian reserve (DOR) is a condition that affects fertility by reducing the reproductive potential of the ovary. The altered expression profile of cumulus cells (CCs) can negatively affect the quality and quantity of oocytes in the ovaries. Recent studies suggest that circulating miRNAs play a significant role in the ovary function, and their serum expression changes can be valuable biomarkers for predicting ovarian function.

**Objective:**

Investigating the expression levels of circulating miRNA-4463 and its target cytochrome P450 19A1 gene (*CYP19A1*) in DOR-CCs in order to find a molecular pathway involved in DOR.

**Materials and Methods:**

In this case-control study, a total of 20 DOR-women and 20 women with normal ovarian reservation aged between 20–34 yr referred to Yazd Reproductive Science Institute, Yazd, Iran were included in the study. Serum and CCs were collected, and real time-polymerase chain reaction was performed to investigate the expression level of miR-4463*, *and its target gene *CYP19A1*.

**Results:**

Our results showed an inverse relationship between miR-4463 and *CYP19A1* expression levels. Therefore, the increase in the expression of miR-4463 was significantly evident in DOR-women compared to the control group (p = 0.0019), while the expression of its target gene, *CYP19A1,* has significantly decreased in these women (p = 0.001).

**Conclusion:**

The present study suggests that miR-4463 and *CYP19A1* pathways could regulate ovary function. Therefore, examination of this miRNA could be a promising parameter for predicting ovarian reserve and their response to stimulation protocols.

## 1. Introduction

Diminished ovarian reserve (DOR) as one of the causes of infertility has recently attracted the attention of researchers (1). Regarding the etiology of DOR, different factors are involved, including genetics, aging, lifestyle, and environment. Evaluating ovarian reservation and personalizing the medicinal plans are necessary for increasing the success rate of assisted reproductive technologies (ART). Failure to respond normally to standard stimulation protocols during in vitro fertilization (IVF) cycles is termed poor ovarian response (POR) (2). Women with POR have a lower implantation rate and increased cycle cancellation rates during IVF compared to age-matched candidates with normal ovarian reservation. Therefore, evaluation of ovarian reserve is necessary to manage infertility treatment in these women (3).

According to the available information, there is no ideal and widespread test to evaluate ovarian reserve and predict response to ovarian stimulation, so more reliable biomarkers are needed (4). Currently, no molecular biomarker, alone or in combination with routine tests, is used to predict ovarian reserve or response to stimulation protocols during ART cycles (5).

MicroRNAs (miRNAs) have recently attracted the attention of many researchers due to their role in various cellular functions. They are an attractive target for use as predictors due to their size, tracking in the body fluids, and convenient identification. Changes in miRNA expression levels have been reported in many reproductive diseases both in males and females (6–12). In women, the role of miRNAs in the development and function of the ovary, in addition to the production of mature and viable oocytes capable of fertilization, development, and implantation, has recently been suggested (13–15).

Whereas circulating miRNAs seem to play a significant role in the ovary function, we hypothesized that changes in their serum expression can be used as potentially valuable biomarkers for predicting ovarian function.

To assess this hypothesis, we conducted a study on DOR-women who were undergoing IVF. Considering the important role of miR-4463 based on the bioinformatics analyses done earlier (16), we aimed to determine whether circulating miR-4463 could be used as a biomarker to measure ovarian reservation and response. In a study, miR-130a-3p, miR-185–5p, miR-329–3p, and miR-4463 were identified to have differential expression in human granulosa cells (KGN) depending on their response to follicle-stimulating hormone (FSH). In this study for the first time in human samples, we studied the expression level of miRNA-4463, to investigate its potential for predicting ovarian response during the IVF process (17). This miRNA has a role in regulating aromatase expression. In aromatase transcriptional regulation, the cytochrome P450 19A1 gene (*CYP19A1*) is the primary gene responsible for producing estradiol (E2) by converting androgens to estrogens and it is one of the gene targets of the aforementioned miRNA (18, 19).

The aromatase enzyme is expressed periodically and specifically in the cumulus cells (CCs) of the ovary, and it is necessary for regulating folliculogenesis, controlling reproductive endocrine glands in women, and coordinating gonadotropin secretion (20).

Any changes in the expression of aromatase enzyme may be effective on ovarian response in DOR-women. In addition, to confirm the role of miRNA-4463 in the ovarian response, the expression of its target gene *CYP19A1* was also measured in CCs.

## 2. Materials and Methods

### Sample collection and ovarian stimulation protocol 

In this case-control study, samples were collected from women referred to Yazd Research and Clinical Center for Infertility, Yazd, Iran from December 2021 to September 2022. The DOR-women group included 20 women aged between 20–34 yr, with an anti-Mullerian hormone (AMH) level of < 1.2 ng/ml and an antral follicle count of < 5 from POSEIDON group 3. Also, among the women referred with male factor etiology, 20 women in the same age range as the DOR-women group but with AMH > 1.2 ng/ml and antral follicle count ≥ 5 were considered as control group. In this research, women with uterine anomalies, endometriosis, polycystic ovary, history of thyroid disease, and chromosomal problems leading to a decrease in ovarian reserve were excluded.

The sampling of DOR-women started with ovarian stimulation using antagonist protocol. On the second day of the menstrual cycle, 300 IU of human menopausal gonadotropin (Merional, IBSA, Lugano, Switzerland) was administrated and the ovarian response was evaluated by performing repeated ultrasounds and determining serum E2 levels. Once the dominant follicle size reached 14 mm, 0.25 mg/day of gonadotropin hormone-releasing hormone antagonist (Cetrotide; Sereno International S.A., Geneva, Switzerland) was injected and continued until the follicle size reached 16–18 mm.

At this time, a human chorionic gonadotropin injection (Pregnyl; Organon, Oss, Netherlands) was performed. After 36 hr of injection, a gynecologist and obstetrician collected samples from the DOR-women. For this objective, the CC sample was collected by the needle connected to the follicular fluid suction device containing the oocyte from the ovarian follicles and the aspirated follicular fluid was poured into a Falcon tube. Moreover, before entering the IVF cycle, blood samples were collected from all participants and their serum was stored.

### Collection and preparation of CCs and serum samples

Oocyte-cumulus cell complexes were collected from the follicular fluid of DOR-women and control group. Also, the CCs around the oocyte were separated using a special needle. Each oocyte was transferred to a glass bottom dish (Wilkos, Netherlands) containing G-Mops-V1 medium (Vitrolife), after washing, and was located in an incubator with 37 C temperature and 6% CO_2_ for 2 hr. Then, 100 µl of the hyaluronidase enzyme (80 IU/ml) (Life Global, USA) was poured into a Falcon tube and placed in an incubator at 37 C for 30 min. The CCs around each oocyte were individually transferred to a Falcon tube and incubated for 15 min. Subsequently, these cells were washed twice using 3 ml of phosphate-buffered saline solution (Inoclon, Iran), and centrifuged at 5000 g for 3 min. The formed precipitate was conveyed to a 1.5 ml RNase-DNase microtube that was previously labeled. In the next step, 200 μl of Tripure solution was added to the microtube and kept in a freezer at -80 C until RNA extraction to investigate miRNA-4463 and *CYP19A1* gene expression. To detect circulating miRNA-4463, whole blood was collected in an EDTA tube before gonadotropin administration from participants of both groups. The samples were centrifuged at a speed of 2500–3000 rpm for 10–15 min and then the clear upper layer (serum) was separated and kept at -80 C until RNA extraction.

### Total RNA extraction and cDNA synthesis

Extraction of total RNA from both CCs and serum of DOR-women and controls was performed using the Tripure RNA Isolation Reagent kit (Roche, Germany) according to its instructions. To check the quantity of extracted RNA, the samples were evaluated by Nanodrop Spectrophotometer (DeNovix, USA) and then stored at -80 C. Bon-Stem miR cDNA Synthesis kit (Stemcellstech, Iran) was utilized to synthesize cDNA for miRNA study. The RevertAid First Strand cDNA Synthesis kit (Thermo Fisher Scientific, USA) was used for total cDNA synthesis, based on the instructions.

### Quantitative reverse transcription polymerase chain reaction (qRT-PCR)

To evaluate the quantitative expression of miRNA and its target gene, qRT-PCR method was performed. SYBR Green quantitative polymerase chain reaction Master Mix 2X AMPLICON kit was used to perform reactions. The thermal profile for qRT-PCR reactions was as follows: 95 C for 3 min, followed by 40 cycles of 95 C for 10 sec, 59 C for 25 sec, and 72 C for 30 sec. In addition, qRT-PCR reactions of the target gene were performed according to the manufacturer's instructions. Moreover, the reactions were performed in duplicates, to avoid possible sampling errors.

The internal reference genes for evaluating the relative expression of *CYP19A1* and miR-4463 were *GAPDH* and U6 snRNA, respectively. The sequence of primers used for evaluating their expression are presented in table I and II. The obtained CTs and the equation 2^-ΔCt^ were used to calculate the expression changes of *CYP19A1* and miR-4463 in the study groups. The serum and CCs of each sample were grouped in the same way and the expression changes of the desired genes were compared between the groups.

**Table 1:** The sequence of primers of U6 and miR-4463 used in the qRT-PCR reactions


**miRNA**	**Sequence (5 ' → 3 ' )**
**miR-4463 forward primer**	AATGAGACTGGGGTG
**U6 snRNA forward primer**	ATCACTGTAAAACCGTTC
**Universal reverse primer**	*
qRT-PCR: Quantitative reverse transcription polymerase chain reaction. *Due to the proprietary nature of the reverse primer purchased from Stemcellstech, its sequence will not be shared with other people	

**Table 2:** The sequence of primers of *CYP19A* and *GAPDH* used in the qRT-PCR reactions


**Gene**	**Primer**	**Tm**	**Product size**
*CYP19A*	Forward: ACACATCTGGACAGGTTGGA Reverse: ATAGCACTTTCGTCCAAAGGG	59	102 bp
*GAPDH*	Forward: CAAGAGCACAAGAGGAAGAGAGAG Reverse: TCTACATGGCAACTGTGAGGA	58.8	103 bp
qRT-PCR: Quantitative reverse transcription polymerase chain reaction, *CYP19A*: Cytochrome P450 family 19 subfamily A member 1, *GAPDH*: Glyceraldehyde-3-phosphate dehydrogenase			

### Ethical considerations

The ethics committee of Shahid Sadoughi University of Medical Sciences of Yazd, Iran, accepted the protocol of the present investigation (Code: IR.SSU.MEDICINE.REC.1400.330). Informed consent was obtained from all participants, and then the sampling was done based on approved guidelines.

### Statistical analysis

The normality of the distribution of quantitative variables was evaluated with the Kolmogorov-Smirnov non-parametric test, considering the skewness and kurtosis indices. To compare the mean of quantitative variables in the 2 studied groups, an Independent *t* test or Mann-Whitney U-test was utilized. Also, the relationship of quantitative variables was evaluated using Pearson's correlation coefficient test. All data were analyzed in SPSS (Statistical Package for the Social Sciences, version 24.0, SPSS Inc., Chicago, Illinois, USA) and Graphpad Prism 9 (Graphpad Software). A significance level of p ≤ 0.05 was considered for all tests.

## 3. Results

### Clinical characteristics of the women with DOR and control group

The demographic information of the participants in the study including their average age and weight (based on body mass index [BMI]) is presented in table III. The average age of the 2 groups is completely matched, but the average BMI of the DOR-women was higher than that of the healthy group. Also, the average hormone levels of FSH, AMH, E2, and LH were measured and compared in both groups (Table III). According to the inclusion and exclusion criteria, the average AMH level in the control and DOR groups was 3.55 ± 0.55 and 0.48 ± 0.09, respectively. Also, the average number of oocytes obtained in the control and DOR groups was 9.86 ± 1.55 and 5 ± 0.71, respectively.

### The results of validating gene targets of miR-4463 

A search was conducted in the miR-Walk database to find potential miRNA targets. This resulted in the identification of 3941 gene targets. Next, the Human Protein Atlas database was searched for all genes involved in ovarian function, and 3841 records were found. Using RStudio software, the intersection of these 2 datasets was determined, validating about 24 gene targets involved in ovarian function (Table IV). The genes were further analyzed using various databases such as The Human Protein Atlas, Ensemble, miRTarBae, and GeneCards to determine their function, location, validated method, and expression status in reproductive-female tissues. Finally, the *CYP19A1* gene, which plays an important role in estrogen metabolism, was selected for further investigation, and its target with miR-4463 was confirmed using 3 different methods based on data from the miRTarBase database.

### Comparison of the relative expression of miR-4463 and *CYP19A1* gene in CCs of DOR-women with healthy women

**Figure 1:**
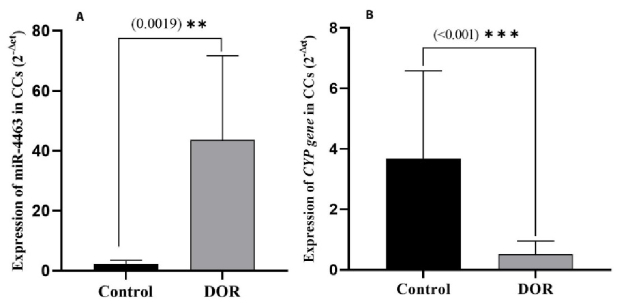
Comparing the relative expression of (A) miR-4463 in the serum of DOR-women with the control group and (B) its target gene (*CYP19A1*) in CCs of DOR-women and the healthy group. DOR: Diminished ovarian reserve. *CYP19A1:* Cytochrome P450 family 19 subfamily A member 1, CCs: Cumulus cells.

The quantitative expression level of miR-4463 in the CCs of DOR-women was measured and compared to women with normal reservation. The results revealed that the relative expression of miR-4463 was higher in DOR-women than in the control group (p < 0.001). On the other hand, the expression level of the *CYP19A1 *gene in the CCs of the DOR-women was compared to that of control group, and it was shown that the expression of *CYP19A1* was considerably diminished in the DOR-women group compared to control group (p < 0.001) (Figure 1).

### Expression changes of miR-4463 and *CYP19A1* gene in CCs of DOR-women regarding the oocyte number

**Figure 2:**
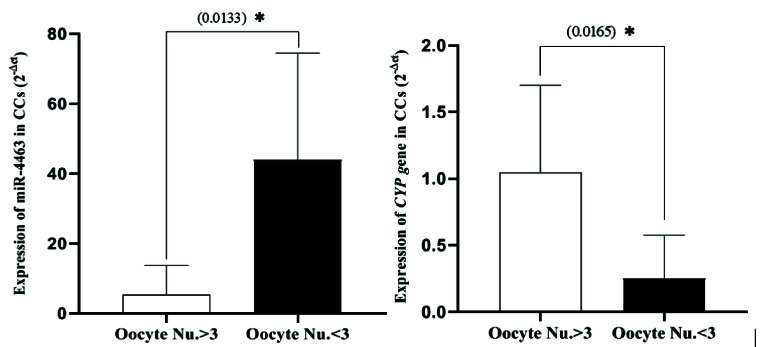
Comparing the relative expression of miR-4463 and *CYP19A1* in 2 subgroups of women with POR: Women with less than 3 oocytes and those with more than 3 oocytes. POR: Poor ovarian response, *CYP19A1:* Cytochrome P450 family 19 subfamily A member 1, CCs: Cumulus cells.

The comparison of the expression of miR-4463 and its target gene (*CYP19A1*) in response to the ovarian stimulation protocol of DOR-women also showed interesting results. In the DOR-women group, the women who had low number of oocytes ( < 3) had a significantly decreased expression of the *CYP19A1* gene compared to women with a normal response ( > 3 oocytes), indicating that poor responders had lower *CYP19A1* gene expression (p = 0.0165). Moreover, it was found that the expression of miR-4463 was significantly higher in poor responders (p = 0.0133). Our findings suggest that the increase in miR-4463 expression had an inhibitory effect on the expression of *CYP19A1*, which may have contributed to the POR in women with DOR (Figure 2).

### ROC curve analysis and comparison of serum level of miR-4463 in DOR-women with healthy women

**Figure 3:**
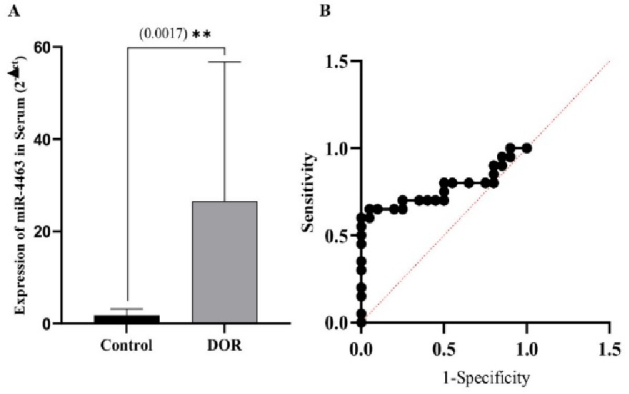
Comparing the serum level of miR-4463 in women with DOR and the control group (A), receiver operating characteristic (ROC) curve analysis using the expression levels of miR-4463 in serum of DOR-women (B). DOR: Diminished ovarian reserve.

To confirm whether the changes in the expression of miR-4463 can be detected in the serum of these women, its serum expression level was also measured. The results showed that the increase in the expression of this miRNA in the serum level of DOR-women compared to healthy women is statistically significant (Figure 3A). The ability of miR-4463 to accurately identify diminished ovarian reservation was measured through the use of receiver operating characteristic (ROC) curves and the area under the ROC curve (area under the curve). The area under the curve values for miR-4463 were 0.76 (p < 0.001), when compared to the serum level of its expression in the control group. The results are depicted as ROC curves of miR-20a and miR-145 based on the serum of DOR and normal reservation women (Figure 3B).

**Table 3:** Demographic and hormonal information of participants


**Variables**	**Control**	**DOR-women**	**P-value**
**Age (yr)**	31.45 ± 2.99	32.6 ± 3.28	> 0.05
**Weight (kg)**	69.95 ± 9.17	66.9 ± 10.55	> 0.05
**FSH (mIU/ml)**	5.46 ± 2.58	5.12 ± 1.16	> 0.05
**AMH (ng/ml)**	3.484 ± 2.43	0.80 ± 0.51	0.0063
**E2 (pg/ml)**	2200 ± 1812	880.78 ± 587.99	0.0380
**LH (mIU/ml)**	1.86 ± 1.06	3.29 ± 2.88	0.0499
**Oocyte number**	9.2 ± 1.55	5.3 ± 3.02	0.0062
Data presented as Mean ± standard deviation, independent *t* test. DOR: Diminished ovarian reserve, FSH: Follicle-stimulating hormone, AMH: Anti-Mullerian hormone, E2: Estradiol, LH: Luteinizing hormone			

**Table 4:** Validated gene targets of miR-4463 involved in ovarian function


**Gene name**	**Open form**	**Chromosome location**	**Gene expression status in female tissues**	**Protein function**	**Validated method**
*CHAC1*	ChaC glutathione specific gamma-glutamylcyclotransferase 1	15q15.1	Vagina > cervix > fallopian tube > ovary	Apoptosis and neurogenesis	NGS
*ABITRAM* *(FAM206A)*	Actin-binding transcription modulator	9q31.3	Fallopian tube > breast > endometrium > ovary	Regulation of actin polymerization	NGS
*BRWD1*	Bromodomain and WD repeat domain containing 1	21q22.2	Placenta > breast > ovary > endometrium	Transcription regulation (cytoskeletal organization)	-
*KY*	Kyphoscoliosis peptidase	3q22.2	Fallopian tube > endometrium > cervix > ovary	Maturation and stabilization of the neuromuscular junction	NGS
*PTPA*	Protein tyrosine phosphatase receptor type A	9q34.11	Placenta > fallopian tube > ovary > endometrium	Negative control of cell growth and division	-
*ASAP1*	ArfGAP with SH3 domain, ankyrin repeat and PH domain 1	8q24.21-q24.22	Placenta > breast > endometrium > ovary	Regulation of membrane trafficking and cytoskeleton remodeling	-
*C5orf22*	Chromosome 5 open reading frame 22	5p13.3	Breast > endometrium > fallopian tube > ovary	Neurofibrillary tangles measurement	NGS
*C9orf64*	Chromosome 5 open reading frame 22	9q21.32	Breast > endometrium > cervix > placenta < ovary	Involvement in the process of the queuosine salvage protein	NGS
*CC2D2A*	Coiled-coil and C2 domain containing 2A	4p15.32	Fallopian tube > ovary > endometrium	Cilia development	NGS
*CDKN1B*	Cyclin dependent kinase inhibitor 1B	12p13.1	Ovary > cervix > breast	Negative control of cell cycle progression in G1	NGS
*EGR3*	Early growth response 3	8p21.3	Ovary > fallopian tube > breast > cervix	Transcriptional regulation and monitoring of biological processes	-
*FRK*	Fyn related Src family tyrosine kinase	6q22.1	Fallopian tube > breast > cervix > ovary	Suppression of cell proliferation	NGS
*GIPC1*	GIPC PDZ domain containing family member 1	19p13.12	Vagina > cervix > breast > fallopian tube > ovary	Regulation of cell surface receptor expression and trafficking	-
*GRHL2*	Grainy head like transcription factor 2	8q22.3	Breast > vagina > fallopian tube > ovary	Primary neurulation and epithelial development	
*INTU*	Inturned planar cell polarity protein	4q28.1	Breast < endometrium > fallopian tube > ovary	Ciliogenesis and embryonic development	NGS
*KIAA0586*	KIAA0586	14q23.1	Ovary > breast > cervix > fallopian tube	Early ciliogenesis	NGS
*MID1IP1*	MID1 interacting protein 1	Xp11.4	Breast > ovary > fallopian tube	Lipid biosynthesis	NGS
*MOGAT3*	Monoacylglycerol O-acyltransferase 3	7q22.1	Placenta > breast > fallopian tube > ovary	Lipid biosynthesis	-
*PLEKHM1*	Pleckstrin homology and RUN domain containing M1	17q21.31	Vagina > cervix > breast > fallopian tube	Autophagy and protein transport	-
*RSBN1L*	Round spermatid basic protein 1 like	7q11.23	Endometrium > ovary > breast > cervix	Dioxygenase activity and metal ion binding activity	NGS
*RTKN*	Rhotekin	2p13.1	Vagina > cervix > breast > endometrium	Involvement in the process of apoptosis	NGS
*TBC1D4*	TBC1 domain family member 4	13q22.2	Ovary > breast > fallopian tube	GTPase activation and rising glucose uptake	NGS
*WNK3*	WNK lysine deficient protein kinase 3	Xp11.22	Ovary > fallopian tube > cervix	Regulation of electrolyte homeostasis, cell signaling, survival, and proliferation	NGS
*CYP19A1*	Cytochrome P450 family 19 subfamily A member 1	15q21.2	Placenta > ovary > breast > endometrium	Estrogen metabolism	Western blot, q-PCR, Micro-array
NGS: Next generation sequencing, q-PCR: Quantitative-polymerase chain reaction, G1: Growth 1 phase, GTP: Guanosine triphosphate					

## 4. Discussion

Due to the significant role of miR-4463 and its target gene, *CYP19A1* in the signaling pathway of steroidogenesis, they were selected for further investigation in our study. Candidate miRNA expression was compared in the participant's CCs and serum, while *CYP19A1* gene expression as its target was measured in their CCs. Our results showed that the expression changes of miR-4463 and its target gene *CYP19A1* in the CCs around the oocyte from DOR-women compared to women were statistically significant. Also, we assessed the serum levels of circulating miR-4463 in 2 groups of normal ovarian reservation (NOR) and DOR women.

Interestingly, in line with the tissue-specific expression changes, the serum level of candidate miRNA was also increased in DOR compared to NOR women. This increased expression of miR-4463 could be one of the reasons why these women had a poorer response to certain ART treatments that involved external gonadotropins. Examining gene expression of *CYP19A1 *in CCs showed a decreasing pattern in DOR-women compared to NOR women. The *CYP19A1 *gene reduces the amount of a hormone called 17β-estradiol in women with reduced ovarian reserve. This decrease in hormone production could explain why some women have a poor response to external gonadotropins. Moreover, to investigate the potential relationship between POR and the candidate miRNA and its target gene, we compared their expression levels between women with 3 or fewer oocytes and those with more than 3 oocytes. Overexpression of miR-4463 and downregulation of the *CYP19A1 *gene in DOR-women with fewer oocytes can indicate their relationship with POR.

Recent research has revealed that miRNA serves to regulate gene expression in various pathological situations, including DOR. Specific miRNAs have been identified in granulosa cells (GCs) of poor responders, which target genes with a role in signaling pathways related to the ovarian response. For instance, miR-106a regulates cell survival and apoptosis of GCs by increasing apoptosis signal-regulating kinase-1 signaling and is shown to have decreased expression in the serum and GC of women with DOR (21). Similarly, miR-21–5p is increased in CCs of POR women and may serve as an important biomarker for oocyte or follicular viability (22). The miR-423–5p is considerably less expressed in high responders than normal responders and is associated with genes such as *CYP19A1, MTHFR, PGR, *and* FSHB*, that are involved in the response to ovarian stimulation (23). Moreover, disruption of miRNA processing leads to changes in ovarian morphology and gene expression. In another study, 4 miRNAs (miR-130a-3p, miR-185–5p, miR-329–3p, and miR-4463) with significant difference in expression were reported. Depending on the response to FSH, they have differential expression in a human granulosa-like tumor cell line, KGN (16). All of these investigations highlight the potential role of miRNAs in regulating follicular growth and function. The CCs have been shown to play a vital role in the development and maturation of oocytes. CCs have bidirectional contact with oocytes during the final stage of oocyte maturation through specialized gap junctions.

Studies have shown various exchanges of data and materials between the CC and the oocyte. When the connections between CC and oocyte are disrupted, it may result in poor oocyte quality leading to weak embryos and pregnancy failure (24). Therefore, examining the gene expression of these CCs can help identify the quality of oocytes and predict the outcome of IVF. In other words, the quality of CCs reflects the quality and even the quantity of oocytes. Therefore, the study of CC genes can be considered as an indicator to check the maturity and competence of oocytes, the growth and quality of the embryo, the outcome of pregnancy and live birth. More importantly, the CCs are isolated through a non-invasive method and discarded during the IVF process. Previously, 2 studies explored the impact of DOR on the transcriptomics of CCs (25, 26).

**Figure 4:**
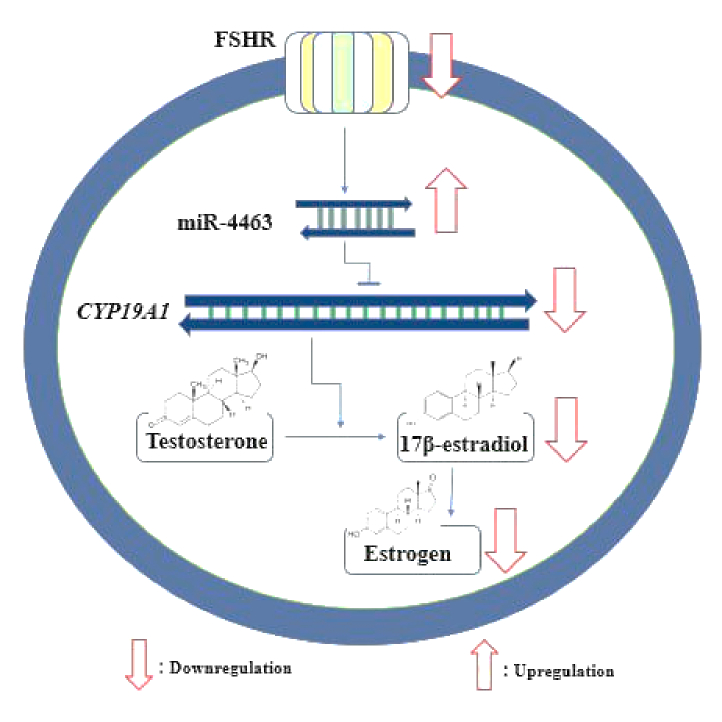
Predicted pathway for the regulatory function of miR-4463 on* CYP19A1 *(aromatase), decreased FSHR expression can affect miR-4463 expression, subsequently, upregulation of miR-4463 in DOR/POR women may result in decreasing *CYP19A1 *gene expression, FSHR: Follicle-stimulating hormone receptor.

Other researchers compared the gene modifications in CCs of women with normal ovarian reserve and those with DOR. Significant advancement has been made in the analysis of CCs transcriptome, with a focus on ovarian reserve status (2). Lee and colleagues discovered 725 differentially expressed genes that were frequently targeted by miR-130a-3p, miR-185–5p, miR329–3p, and miR-4463 using different bioinformatics tools. 2 of those genes were confirmed including *CYP19A1* and *ESR1*. Specifically, *CYP19A1* encodes the aromatase, which is crucial for synthesizing 17β-estradiol in GCs. The research team suggested that in KGN cells, significantly reducing the protein product level of *CYP19A1* is the target of miR-4463. This advocates that 17β-estradiol synthesis is regulated by miR-4463 through targeting *CYP19A1* (16). Another study team hypothesized that the increased expression of miR-4463 in the serum of women with polycystic ovary syndrome and DOR may be related to low response and decreased estrogen production. This is due to a report of reduced aromatase activity in PCOS follicles caused by *CYP19A1* genetic polymorphism (Figure 4).

In IVF cycles, predicting ovarian response to external gonadotropins is crucial, and personalized medicine can improve treatment management for these women. Considering the importance of finding a marker to anticipate the response to stimulation protocol in ART, by citing the previous bioinformatics and experimental studies, miR-4463 and its target gene *CYP19A1* were selected for investigation (16). We searched the database to find potential miRNA targets. This resulted in the identification and validation of about 24 gene targets involved in ovarian function. The genes were further analyzed using various databases to determine their function, location, and expression status in reproductive female tissues. Finally, the *CYP19A1* gene, which plays an important role in estrogen metabolism, was selected for further investigation. In the present study, we compared the expression changes of this miRNA and its target gene in CCs of normal ovarian reserve and DOR individuals.

Numerous studies have examined miR-4463 in vascular conditions, for example, atherosclerosis and carotid artery stenosis (27, 28). High levels of miR-4463 increase oxidative stress due to H_2_O_2_ induction, which leads to apoptosis in endothelial cells (29). On the other hand, inhibiting miR-4463 enhances the survival of human umbilical vein endothelial cells and reduces apoptosis under a hypoxic state by targeting the nuclear protein phosphatase-1 subunit (30). Moreover, miR-4463 promotes the migration of vascular smooth muscle cells, which is associated with vascular disorders (31). It is noteworthy that miR-4463 expression is significantly elevated in women with PCOS, but the biological functions associated with PCOS are not yet understood (32).

## 5. Conclusion

Our research suggests that measuring the serum level of circulating miR-4463 can provide a dependable way to predict the likelihood of a woman having a poor response to a specific stimulation protocol in ART cycles. Nevertheless, to attain more accurate predictions of ovarian response, it is crucial to replicate this investigation on a larger sample size. It is also suggested to investigate other target genes that play a role in ovarian function and some of them are mentioned in this article.

##  Data availability

The data supporting the findings of this study, are available from the corresponding authors upon reasonable request.

##  Author contributions

A. Yazdanian and MH. Sheikhha designed the study and conducted the research. A. Yazdanian, M. Lotfi, and F. Montazeri monitored, evaluated, and analyzed the results of the study. Further, S. Dashti, A. Yazdanian, and F. Montazeri reviewed the article. All authors approved the final manuscript and take responsibility for the integrity of the data.

##  Conflict of Interest

The authors declare that there is no conflict of interest.
